# Clinical Diagnosis and Treatment of Ruptured Renal Artery Aneurysm During Pregnancy: A Case Report and Literature Review

**DOI:** 10.1002/ccr3.72820

**Published:** 2026-06-12

**Authors:** SuFang Qiu, Yao Xiao, Yan Chen, Minglin Zhong

**Affiliations:** ^1^ Department of Obstetrics Shaoguan Maternal and Child Health Hospital Shaoguan China; ^2^ Department of Reproductive Medicine Center Shaoguan Maternal and Child Health Hospital Shaoguan China; ^3^ Department of Guangdong Cord Blood Bank Guangdong Women and Children Hospital, Guangzhou Guangdong People’s Republic of China

**Keywords:** diagnosis, pregnancy, renal artery aneurysm, rupture, treatment

## Abstract

Rupture of renal artery aneurysm during pregnancy (PSRRAA) is a life‐threatening emergency that can lead to adverse outcomes for both the mother and the fetus. This paper reports a case of right renal artery aneurysm (RAA) rupture in a 32‐year‐old pregnant woman at 37 weeks of gestation, and discusses the clinical diagnosis and treatment of renal artery aneurysm rupture, aiming to provide reference and guidance for subsequent diagnosis and treatment of this disease.

## 1 Introduction

Renal artery aneurysm (RAA) refers to an abnormal bulging and dilation of the main trunk or branches of the renal artery, which is a rare visceral aneurysm [1]. Clinical studies have shown that pregnancy is one of the high‐risk factors for RAA rupture. RAA rupture occurring during pregnancy and the perinatal period may be masked by labor pain and easily confused with obstetric emergencies, such as placental abruption, which requires high vigilance [2]. The risk of RAA rupture during pregnancy is high, and in severe cases, it may even endanger the health and life of both mother and fetus. Therefore, timely diagnosis of RAA rupture is crucial for improving the therapeutic effect of the disease. However, the symptoms and signs of RAA lack specificity, which to a certain extent increases the difficulty of disease diagnosis, affects the accuracy of diagnosis, and complicates diagnosis and treatment [3].

Epidemiological studies indicate that the incidence of RAA in the general population ranges from 0.01% to 0.5%, with rupture occurring in only approximately 0.09% of patients with RAA [4]. The occurrence of RAA during or before pregnancy is rare, and pregnant women with combined RAA rupture are even rarer. Available evidence suggests that following RAA rupture and hemorrhage in pregnant women, the maternal mortality rate can be as high as 92%, the rescue survival rate is approximately 65.6%, and the perinatal survival rate is 40.6%, underscoring the critical nature and poor prognosis of this condition [5].

This study analyzes a patient who underwent emergency lower uterine segment cesarean section combined with transcatheter right renal artery angiography and embolization due to “1. 37 weeks of gestation with mature fetus; 2. Acute rupture and hemorrhage of right renal artery aneurysm with persistent decrease in hemoglobin,” aiming to explore the clinical diagnosis and treatment of ruptured RAA and provide reference for subsequent clinical practice.

## 2 Key Clinical Message

Ruptured renal artery aneurysm in pregnancy is life‐threatening. At term, emergency cesarean section plus endovascular embolization can be lifesaving. However, if recurrent bleeding, renal infarction, or infection occurs, timely nephrectomy is crucial to ensure maternal and fetal survival.

## 3 Case Presentation

A 32‐year‐old female, gravida 3, para 2, with a history of two full‐term vaginal deliveries (two daughters born in 2017 and 2019), was admitted to the hospital at 10:47 on May 16, 2024, due to “37 + 1 weeks of gestation and right lumbabdominal pain for more than 9 hours”. The patient had unremarkable prenatal examinations throughout pregnancy and presented with recurrent cough over the past week. At 1:00 on May 16, she developed right lumbabdominal pain without obvious inducement, accompanied by mild lower abdominal pain, but no vaginal bleeding or fluid discharge. She was admitted to the outpatient department with a preliminary diagnosis of “abdominal pain (renal colic?)”. On admission, vital signs were stable; physical examination revealed significant tenderness in the right costovertebral angle and mild tenderness in the right lower abdomen without rebound tenderness or muscular rigidity, and the abdomen was distended consistent with gestational age.

**FIGURE 1:**
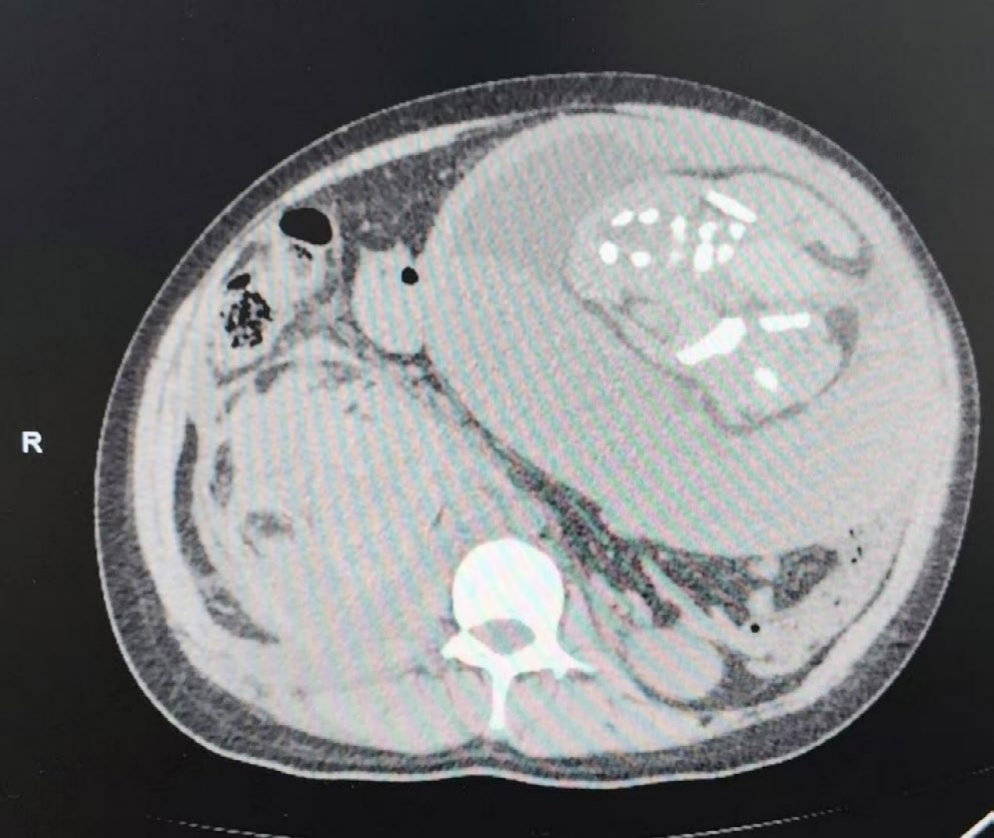
Ruptured right renal artery aneurysm with hemorrhage.

Auxiliary examinations showed a normal fetal color Doppler ultrasound on May 15 and a hemoglobin level of 76 g/L in routine blood tests. Coagulation function, preoperative eight‐item screening, liver and kidney function were unremarkable after admission. Urinary system color Doppler ultrasound indicated multiple small stones in the right kidney, a 69 mm × 159 mm × 85 mm hypoechoic mass in the right abdomen (ill‐defined boundaries, heterogeneous echo), and a small amount of ascites. Emergency abdominal contrast‐enhanced CT (Figure 1, performed at another hospital) confirmed right renal artery aneurysm rupture complicated with retroperitoneal and abdominopelvic hematocele, bilateral renal cysts, and a single intrauterine fetus in cephalic presentation. Emergency routine blood tests showed leukocytes 19.78 × 10^9^/L, red blood cells 2.9 × 10^12^/L, and hemoglobin 50 g/L.

Due to “mature fetus at 37 + 1 weeks of gestation and acute right renal artery aneurysm rupture with persistent hemoglobin decline,” the patient underwent emergency lower uterine segment cesarean section combined with transcatheter right renal artery angiography and embolization. Intraoperatively, a large purple‐blue hematoma with high tension was observed in the retroperitoneum extending from the right broad ligament to the right pelvic wall. Interventional angiography revealed a rupture at the ostium of the second‐order branch of the upper pole of the right renal artery with pseudoaneurysm formation. After failed superselective embolization, six tower‐shaped coils were implanted at the distal main trunk of the right renal artery to achieve complete occlusion. The operation was uneventful with an estimated blood loss of 200 mL; intraoperative transfusion included 6 units of B‐type Rh‐positive suspended red blood cells, 1200 mL of plasma, and 5 units of cryoprecipitate, with postoperative hemoglobin increased to 62 g/L. The final diagnoses were right renal artery aneurysm rupture, gravida 3 para 2, 37 + 1 weeks of gestation, LOA, cesarean section with a live singleton infant, severe anemia, thalassemia, and neonatal mild asphyxia. The patient was transferred to the ICU for monitoring after surgery.

On the first postoperative day, 600 mL of pale bloody fluid was drained from the pelvic drainage tube, and hemoglobin progressively decreased to 51 g/L; two additional units of suspended red blood cells were transfused (hemoglobin increased to 57 g/L), along with anticoagulation with nadroparin calcium and infection prevention with cefuroxime. On the second postoperative day, the patient's condition stabilized with 140 mL of drainage in 24 h, and she was transferred to the obstetric ward. Another two units of suspended red blood cells were transfused (hemoglobin increased to 70 g/L), and a follow‐up CT showed slightly increased retroperitoneal hematocele with increased density. On the third postoperative day, the patient developed fever with elevated inflammatory markers (procalcitonin 0.948 ng/mL, interleukin‐6275.97 pg/mL, C‐reactive protein 33.94 mg/dL). Repeat abdominal contrast‐enhanced CT revealed right renal enlargement with multiple hypo/non‐enhanced areas (suspected renal infarction). Considering recurrent rupture of the right renal aneurysm and hematoma complicated with infection, the patient underwent emergency right nephrectomy, retroperitoneal hematoma evacuation, and adhesiolysis. Intraoperatively, approximately 1000 mL of hematoma and hematocele were cleared with an estimated blood loss of 200 mL; two units of suspended red blood cells and 600 mL of virus‐inactivated plasma were transfused.

Postoperatively, the patient was transferred back to the ICU for anti‐infection therapy with cefoperazone‐sulbactam and thromboprophylaxis with nadroparin calcium. Subsequent follow‐up examinations showed gradual reduction of retroperitoneal and abdominopelvic hematocele, as well as improvement of pulmonary atelectasis/inflammation and pleural effusion. On the 12th postoperative day, the patient's general condition was good with normal bowel and bladder function. Follow‐up laboratory tests showed leukocytes 9.32 × 10^9^/L, hemoglobin 70 g/L, procalcitonin 0.406 ng/mL, and interleukin‐6 17.31 pg/mL, and she was discharged successfully.

## 4 Discussion

RAA is a local cystic or fusiform dilation of the renal artery, exceeding 50% of the diameter of the adjacent artery [6]. Previous clinical studies have shown that pregnant women are a high‐risk group for RAA rupture. Analysis of the etiology of RAA shows that the disease has complex causes. Relevant scholars have indicated that it is directly related to hypertension, atherosclerosis, Ehlers‐Danlos syndrome, and fibromuscular dysplasia [7, 8]. In general, RAA grows slowly and can grow naturally for up to 270 months without rupture, with an annual diameter increase of 0.06–0.60 mm. Common risk factors for RAA rupture include hypertension, pregnancy, solitary kidney, progressive enlargement of the aneurysm, incomplete calcification of the aneurysm, and diameter > 2 cm. For female RAA patients, childbearing age is a common indication for treatment. By analyzing the reasons for the high incidence of RAA rupture during pregnancy, relevant scholars believe [9] that abdominal surgery, childbirth, atherosclerosis associated with advanced age, increased blood volume, irreversible vascular damage caused by multiple pregnancies, increased abdominal pressure, increased renal blood flow during pregnancy, clockwise displacement of the enlarged uterus compressing the right renal artery, and weakening of blood vessel walls by pregnancy hormones are high‐risk factors for RAA rupture.

Clinical studies have shown that to improve disease treatment and prognosis, attention should be paid to the identification and management of RAA during pregnancy [10]. Since most diseases have no obvious symptoms after occurrence, some patients may experience low back pain, hematuria, uncontrollable hypertension, abdominal pain, etc., accounting for about 25% according to data. It is easy to be confused with kidney stones, placental abruption, threatened preterm labor and other diseases, leading to misdiagnosis and delayed treatment [11, 12]. After RAA rupture, hemodynamic instability of the patient will occur, increasing the risk of hemorrhagic shock and endangering life safety. However, due to factors such as increased abdominal pressure of pregnant women and compression by the fetus in the abdomen, the difficulty of disease diagnosis increases [13]. In this case, after admission, urinary system color Doppler ultrasound showed normal size and shape of both kidneys, smooth capsule, uniform parenchymal echo, and no obvious separation of the collecting system. No obvious abnormal echo was found in the left kidney. Scattered punctate strong echoes were seen in the right kidney without obvious posterior acoustic shadow. The vascular distribution in both kidneys was normal. Emergency CT examination suggested: 1. Enlargement of the right kidney, multiple exudates and hematomas around the right kidney and retroperitoneum, considering the possibility of spontaneous rupture of the right kidney, further examination was recommended; 2. Multiple small stones in both kidneys; 3. Single intrauterine fetus, cephalic presentation. Combined with clinical manifestations, spontaneous renal rupture was considered. At this time, the condition was more difficult to judge, and it was easy to be confused with infectious shock caused by renal abscess and infectious shock associated with kidney stones. For better disease diagnosis, clinical differentiation can be carried out according to imaging diagnosis results, infection indicators, and the patient's disease course [14].

At present, there are many clinical diagnostic measures for RAA, including computed tomography (CT), magnetic resonance angiography (MRA), ultrasound, and digital subtraction angiography (DSA) [15]. In this case, diagnosis was made through whole abdominal CT plain scan + enhancement and DSA, which had high guiding value for treatment. CT can perform tomographic scanning of the human body using radiation, with high resolution for lesions, and can make disease diagnosis under three‐dimensional reconstruction, facilitating the implementation of subsequent diagnosis and treatment. However, this measure has limitations such as contrast medium toxicity and radiation, which to a certain extent limits its application. DSA can clearly show the distribution of blood vessels, has high diagnostic accuracy, and is helpful for the diagnosis and treatment of various cerebrovascular diseases. It has high image quality with high definition and accuracy, can clearly show the fine structure of blood vessels, provide real‐time guidance, and has the characteristics of minimal invasion, less patient pain, high acceptance, fast recovery, strong targeting, good compatibility, and can provide more comprehensive diagnostic information. The patient in this case had unstable hemodynamics and critical condition. According to the actual situation, emergency rescue was carried out. Through whole abdominal CT plain scan + enhancement and DSA, the possibility of RAA rupture was identified, and right renal artery angiography and embolization were performed. The above analysis indicates that in subsequent clinical diagnosis, whole abdominal CT plain scan + enhancement and DSA can be fully utilized, and attention should be paid to improving ultrasound examination to better provide reference for disease diagnosis and treatment. The Clinical Practice Guidelines for the Management of Visceral Aneurysms by the Society for Vascular Surgery suggest that there is a direct correlation between RAA and fibromuscular dysplasia. The arterial lesions of patients with fibromuscular dysplasia include focal or multifocal types. Pregnant women with combined RAA should be screened for fibromuscular dysplasia, which can be evaluated by magnetic resonance imaging or CT angiography. The patient in this case was successfully discharged after effective diagnosis and treatment. Postoperatively, anticoagulant therapy with “nadroparin calcium” and preventive anti‐infection therapy with “cefuroxime, cefoperazone sulbactam” were given. “According to the 2021 Chinese expert consensus on the prevention and treatment of VTE during pregnancy and the puerperium, prophylactic low‐molecular‐weight heparin (LMWH) is recommended after cesarean section and following severe postpartum hemorrhage or massive blood transfusion, once active bleeding has been excluded. Our patient was at high risk for thrombosis (cesarean delivery, massive transfusion, immobilization, severe anemia); thus, anticoagulation with nadroparin calcium was initiated on the first postoperative day. However, given the patient's subsequent clinical course—recurrent hematoma and eventual nephrectomy—we recognize that the optimal timing of anticoagulation in such critically ill patients remains challenging and should be individualized, weighing the risks of thromboembolism against the risk of re‐bleeding.” Nadroparin calcium can promote endothelial cells to release anticoagulants, mainly has an anticoagulant effect, with strong antacid effect and long duration of action, and is mainly used for the prevention of thromboembolic diseases. Cefuroxime can inhibit the synthesis of bacterial cell walls, inhibit cell division and growth, and ultimately lead to bacterial lysis and death, which is a second‐generation cephalosporin antibiotic. Cefoperazone sulbactam and amoxicillin clavulanate potassium can enhance the antibacterial activity of the drug, improve inflammatory lesions caused by sensitive bacterial infections, and achieve anti‐inflammatory and bactericidal effects. The above medications can help patients effectively inhibit infection and have positive significance for accelerating the disease recovery process.

Comparison with splenic artery aneurysm rupture in pregnancy. Splenic artery aneurysm (SAA) is the most common visceral artery aneurysm in pregnancy, with approximately 78% of cases occurring during the third trimester [16]. The incidence of SAA in the general population ranges from 0.01% to 0.2% [17]. Similar to RAA, pregnancy is a recognized risk factor for SAA rupture due to physiological changes including increased blood volume, increased cardiac output, hypertension, and hormonal influences that weaken the arterial wall [18]. Barranco et al. reported a case of sudden SAA rupture in a 26‐year‐old primigravida at 27 weeks of gestation, resulting in fetal death but maternal survival; histological examination revealed intimomedial mucoid degeneration of the splenic artery [16]. The maternal mortality rate for ruptured SAA in pregnancy is approximately 75%, with fetal mortality as high as 95% [19]. In contrast, ruptured RAA in pregnancy carries an even higher maternal mortality rate of up to 92% and a perinatal survival rate of only 40.6% [5]. From a diagnostic perspective, RAA rupture typically presents with flank pain, whereas SAA rupture more commonly presents with epigastric or left hypochondrium pain, sometimes radiating to the left shoulder (Kehr's sign) [16]. Both conditions are easily misdiagnosed as obstetric emergencies such as placental abruption, uterine rupture, or appendicitis [20]. Ultrasound with Doppler is the preferred initial imaging modality due to its non‐invasive nature and absence of fetal radiation exposure, but CT angiography remains the gold standard for definitive diagnosis when clinical suspicion is high [21]. Management principles for both conditions emphasize prompt hemodynamic stabilization and expeditious surgical or endovascular intervention. For SAA, treatment options include transcatheter embolization, laparoscopic ligation, or splenectomy, whereas for RAA, endovascular coiling or embolization is often feasible when anatomy permits [22]. In cases of rupture with hemoperitoneum, emergency cesarean section followed by laparotomy and aneurysm repair or nephrectomy/splenectomy is necessary to save maternal life [23]. Awareness of the similarities and differences between these two visceral artery aneurysms may aid clinicians in the differential diagnosis of acute abdominal pain in pregnancy and guide appropriate management.

Due to the risk of RAA rupture during pregnancy, the clear indication for the treatment of RAA during pregnancy is RAA > 1.5 cm in pregnant women or women of childbearing age. The Clinical Practice Guidelines for the Management of Visceral Aneurysms by the Society for Vascular Surgery (SVS) also point out that women of childbearing age with RAA with or without complications and at surgical risk should receive surgical treatment regardless of the size of the RAA tumor. If symptoms or rupture occur, emergency intervention should be performed [24]. For the repair of RAA during pregnancy, surgery is the main clinical treatment plan. Relevant studies have shown that for pregnant women with suspected or confirmed RAA and unstable hemodynamics, laparotomy should be performed, followed by RAA resection, nephrectomy, and cesarean section, but the gestational age was not discussed [25]. At present, the optimal surgical method for RAA is still controversial, which can be selected individually according to the condition and type of RAA. Open aneurysm repair can reconstruct the artery with relatively large trauma, but the long‐term patency rate of the renal artery after surgery can reach 93%–100% [26]. For pregnant women and other high‐risk groups, partial or total nephrectomy is feasible in cases of renal aneurysm rupture with hemodynamic instability. Nowadays, endovascular interventional surgery has developed rapidly in the treatment of RAA, with short recovery time, low risk of death, and high success rate, and has been widely used in the treatment of visceral aneurysms.

In conclusion, the risk of renal artery aneurysm rupture is high, and it is easy to be confused with other pregnancy complications. The condition is dangerous. Clinical attention should be paid to disease diagnosis and treatment. Various diagnostic measures should be fully utilized to achieve early diagnosis, identification, and treatment of the disease. Close follow‐up and timely intervention should be noted to maximize the rescue of maternal and child lives.

## Author Contributions

**SuFang Qiu:** software, supervision. **Yao Xiao:** resources, software, writing – original draft. **Yan Chen:** conceptualization, data curation, resources, software. **Minglin Zhong:** data curation, resources, software.

## Funding

The authors have nothing to report.

## Consent

Written informed consent was obtained from the patient for publication of this case report and accompanying images. A copy of the consent form is available for review by the Editor‐in‐Chief of this journal.

## Conflicts of Interest

The authors declare no conflicts of interest.

## Data Availability

All data underlying this case report are included within the article. Additional clinical information is available from the corresponding author upon reasonable request, in accordance with patient privacy regulations.
